# Book Reviews

**Published:** 1805-04-01

**Authors:** 


### CRITICAL ANALYSIS
### OF THE
### RECENT PUBLICATIONS
### ON THE
### DIFFERENT BRANCHES OF PHYSIC, SURGERY, AND MEDICAL PHILOSOPHY.

---

*Observations on Cancer, connected with Histories of the Disease.* By Everard Home, Esq. F. R. S. Surgeon to St. George's Hospital. London. 8vo. pp. 248

In an Introduction the author obeserves, that, notwithstanding the destructive nature of cancer, it still remains not only an incurable, but a disease not well defined. This is certainly most deplorably true, and it is with much pleasure we find the subject at last seriously taken up by regular practitioners.

The late Mr. Justamond led the way with a zeal which was supported by sanguine hopes of discovering a cure. Though in this he failed, he has very much served the cause in which he engaged, by a candid acknowledgement of his failure, and an accurate history of the manner in which he gave so many remedies a fair trial. Mr. Pierson followed with "Practical Remarks on Cancer," which, like all that Gentleman's writings, contain a large collection of authorities as well as many useful remarks. Dr. Adams, in his "Observations on Cancerous Breasts," has assumed the bolder task of forming a theory, or at least, a division, of the different species of cancer to which those glands are liable. To what extent he has succeeded cannot easily be ascertained, till we have a much larger accumulation of facts than the present state of the science furnishes. We must, however, allow him the credit of having stimulated the industry of others. To his publication we probably owe Mr. Abernethy's attempt at a classification of tumours, and also the work now before us.

Mr. Home, after regretting that so little has been done towards an accurate investigation of this most painful and fatal malady, goes on to remark, that the most rational mode of diffusing the necessary knowledge is, that each practitioner should lay lay the result of his inquiries before the public, in order to form a fund of accurate observations. Perhaps this is the best means that can be proposed, considering those jealousies which must ever exist among men engaged in the same pursuit. But there cannot be a doubt, that if mutual communications were made to each other, and mutual opportunities offered of seeing each other's cases, a much more correct knowledge would be formed; and every work, before it met the public eye, would acquire a degree of accuracy we cannot otherwise expect. Till this desirable event is accomplished, we shall receive with pleasure, and review with candour, every contribution, however imperfect, on this important subject.

Chapter I. contains two "Cases of Cancer, the Origin of which was ascertained." The first subject was a sailor, who four years before his admission into the hospital, by a fall from the main-mast, had reduced the glans penis to a perfectly flat figure. By rest and proper treatment the part recovered its natural form, excepting a small pimple on the extremity, which, six months afterwards, became a painful ulcer.—The symptoms increased, and were further exasperated by a mercurial course. For two months afterwards a variety of remedies were tried in the Lock Hospital, during which the disease gradually gained ground, till he was received into St. George's Hospital by Mr. Home. "At this time, we are told, the glans penis had the appearance of a cancerous tumour, with a small deep ulceration in the anterior part of it; and all round a tuberculated margin forming a thick ridge, partly covered with a thin cuticle, which the author adds, is an appearance I consider as peculiar to cancer." Arsenical remedies were exhibited in poultice, and internally; but with no advantage. The disease continued to increase under every mode of treatment, till the groins partook of the ulceration, fungus, and occasional hæmorrhage; the lower limbs were œdematous; hectic sometimes supervened; the pain and extent of the disease were only arrested in one part, to spread with more rapidity in another; and, in the end, the patient died miserably.

"On opening into the cavity of the abdomen, the peritoneum, which lines the abdominal muscles between the pubes and the navel, was studded over with small white opake bodies, of a firm consistence. Immediately within the abdominal ring, the lymphatic glands were in a diseased state: on the left side there were two of the size of chesnuts; and on the right there were three of the same size: a chain of diseased glands, of different sizes, was traced from these round the margin of the pelvis to the lumbar vertebræ. On the anterior surface of these bones they formed a solid mass, an inch and a half in thickness, completely surrounding the aorta and vena cava. They were met with as high as the sixth dorsal vertebræ, gradually diminishing in size and number to that part; but none could be discovered on the anterior part of the spine, higher up.

"There

"There was a large cluster of similarly diseased glands at the root of the mesentery, and a few were met with in the mesentery.

"The psoas muscle of the left side, in which the diseased glands were in the greatest number, was studded over by small oval white bodies of a firm consistence, not much larger than millet seed, the interstices between them not exceeding one-eighth of an inch. On cutting into the substances of the muscle, the interstices between the fasciculi of muscular fibres contained a number of the same substances: they appeared to be evidently connected with the diseased state of the lymphatic system.

"These glands, when cut open, presented different appearances; those nearest the groins contained a soft white substance, of the consistence of thick cream; and those higher up in the loins were harder and more solid.

"The liver was studded over on both its surfaces with flattened hard irregular bodies of different sizes, from that of a silver penny to that of a shilling; their internal substance, when cut into, was similar to that found in the glands above described,

"The other viscera contained in the cavity of the abdomen, had their natural appearance.

"Upon examining the thorax, the lungs were found in a healthy state; the heart was rather smaller than it is usually met with. On the anterior part of the diaphragm there were several tumours of the size of chesnuts; and on different parts of the pleura, in both sides of the chest, smaller tumours were met with; all of these resembled, in their internal texture, the diseased glands in the loins.

"I have two cases of cancer of the penis now under my care, the progress of which is very similar to that above described. In both of these the disease began in the prepuce, and extended itself to the glans, which is by far the more common origin of the disease. Mr. Hey (in his Practical Obervations in Surgery, illustrated with Cases) has given so many accurate histories of cancer of the penis, arising from this cause, as to make any addition to them superfluous."

On this important case we shall make no remarks, but that, in the description of the appearances after death, we confess we expected from Mr. Home a more accurate account of these tumours. In some places we read of tumours, in others of diseased glands, and, lastly, we are told that the tumours resembled the diseased glands. Now we shall be glad to be informed, what proofs there are that any of them were glands, or that they were not all such? Among the old physiologists, it was customary to call every appearance of this kind a gland; but our readers will see, that if a gland is so much altered as to become a tumour containing a soft white substance of the consistence of thick cream, it will be difficult to say what the original structure may have been, or whether the whole were new-formed parts.

The

The second case was in the foot, and either arose from, or was first discovered in consequence of, wearing a tight shoe. The mischief continuing to increase for a great length of time, the limb was at length amputated. Though the diseased action had extended to all the surrounding parts, it does not appear that the glands were affected. The tumour is said to have "the striated texture which characterizes the disease." We sincerely wish to have been informed, whether by *the disease* is here meant cancer. We acknowledge there are often tumours of such a description as are not easily described; but in that case, we wish the account not to be so entirely unembarassed.

Chapter II. contains cases of tumours in the breast, which were indolent in their origin, and became afterwards true cancers. The three first cases were removed during their indolent state. But the author conjectures they might have proved cancerous by the result of the three following ones. The similarity is not easily ascertained. However, those who wish to compare them with each other, and with cases which have occurred in their own practice, must consult the work itself.

Chapter III. contains "Cases of Cancer of the Breast, which illustrate many of the Symptoms of the Disease." The first was the effect of a blow at the early age of fifteen. The author had not an opportunity of seeing the conclusion of the case, but we regret much that he has given no description of the tumour after it was removed.

The inference we should draw from the next case would be, that whenever the operation is performed, it is best to remove the whole breast; and where the glands of the axilla show the disease, to remove also as much of them as the knife can reach. This woman submitted to the operation four times. The texture of the first tumour is not described.

We have next a case of cancer in the breast, in which the lymphatic glands towards the clavicle were the only ones contaminated. This affords no practical remarks, as the patient is still living, and none of the diseased parts has been removed by art.

The following case is of cancer in the breast attended with swelling in the arm, from the effects of the disease on the glands in the axilla. These cases are very common; but we could wish, that before this time, the nature of this swelling in the arm were better understood. We well know that the lymphatic glands and vessels are so universally diffused, and anastomose so universally, as to keep up absorption after the destruction of many of them.

In the next case the swelling of the arm did not take place till after the operation, and by its increase produced so much pain as to terminate the life of the patient. It appears that the disease had extended to the ribs and muscles of the chest.

The next case affords no practical hints, excepting that it spread (as the author supposes by the glands) towards the sternum.

In the following case, however, small tumours were produced over over a considerable part of the body by the contamination of the skin.

In the sixteenth case a tumour was extirpated, and during the healing process the skin appeared contaminated; but by destroying the parts the patient continued free from the disease. We have no account of the tumour taken out.

In the succeeding case the disease had affected the pectoral muscle before the operation was performed. Afterwards an appearance followed, which very much resembled that disease so well described by Mr. Hays under the name of Fungus Hæmatodes. We have again to regret that no account is given of the appearance of the tumour first extirpated.

In the eighteenth case the disease extended over the lungs in such a degree as to prove fatal. Tumours of different kinds were found, which the author considers as the lymphatic glands of the lungs.

In the nineteenth case considerable inflammation attended the disease in consequence of a journey. In the event, it appeared that this inflammation was not cancerous.

The twentieth case was attended with pain and swelling in the arm, without any apparent disease in the axillary glands. This should at least teach us to doubt whether swellings of this kind are ever occasioned by obstructions in these glands.

In the succeeding case the principal pain experienced by the patient was found by the operation to arise from the pressure of the tumour on the nerve; a similar instance is related in an abscess by the knee.

A case follows, in which the cancerous tumour mortified and was cast off, but the local disease afterwards spread, and other parts about the body began to mortify.

The succeeding case contains nothing but what often occurs.

The next Chapter contains two cases of Hydatids in the Breast. The first, Mr. Home supposes arose from blood originally effused and coagulated. Being afterwards absorbed, the surrounding parts were too compact to collapse and fill up the space, in consequence of which a watery fluid was deposited. This, it is added, is *exactly* similar to what happens in the brain. We would gladly know how our author acquired this *exact* information? From this and the succeeding case, and also from the frequent occurrence of these cysts in cancerous breasts, Mr. Home very rationally supposes that hydatids are not necessary to the existence of cancer, but accidentally found in those tumours. This appears to be Dr. Adams's opinion of what he calls the lymphatic hydatid, or watery incysted tumour.

Two cases follow of cancerous tongues, which proved fatal without any operation. In the section of one of them after death, immediately under the coronal process of the lower jaw was situated a hard and conspicuous tumour, which, on examination, appeared to be a gland weighing two ounces and a half, measuring an inch inch in thickness, and a trifle more in diameter. We much regret that we are not instructed how we are to distinguish what are really glands in this altered state. The thyroid gland was diseased, but we are not informed whether it had the same appearance as the enlarged one above-mentioned.

A Chapter follows of cases, which illustrate the symptoms of cancer in the testicle. These cases are all valuable, and the description of the original tumour in all, and in most of those in other parts of the body after death, are given, we doubt not, with accuracy, but not with sufficient minuteness. In one case we have an account of the greater part of the body being filled with diseased lymphatic glands. On cutting into them, it was found that "those which had not arrived at suppuration contained a substance, not unlike soft cream cheese; those further advanced, appeared to be only a bag containing a whitish matter tinged with blood, and some nearer the pubes consisted of a white thin matter like cream with no tinge of red whatsoever." In another case, the diseased part, which was removed, consisted only of a thickened tunica vaginalis filled with firm coagulum. On examining the body after death, there were found large masses of swellings, not much firmer than strong coagulated milk with the whey in it. These masses extended up the left side along the back; on the epiploon was a large mass connecting the colon, stomach, and other viscera together. The liver was studded with similar tumours. We presume they were enclosed in some sac, but that is not described. There seems to have been no suspicion that these were lymphatic glands.

Two cases follow of cancer in the rectum, from which, and the author's observations on other cases of cancerous intestines, he remarks that he has never found the absorbents affected from these causes. This would lead us to suspect that such diseases are essentially different, though equally incurable. Some good practical remarks follow.

We have next two cases of the disease which Mr. Hunter called a fungated sore. As amputation of the limb was performed in both, it is to be regretted that no account is given of the appearances of the limb.

A long Chapter follows "on the Nature and Progress of Cancer," which is as full of alternate description, conjecture, proposals, and caution, as are usually met with in most authors on this subject. Some remarks succeed on the different modes of extirpating cancers, in which the author shows that the actual cautery is by no means so formidable as it appears to be. He however prefers the knife on most occasions, and offers some useful remarks on the mode of extirpating cancerous tumours from the breast by that instrument. The paper read before the Royal Society on the extirpation of tumours from the tongue by ligature is somewhat enlarged; and the work closes with remarks on the mode of extirpating by the knife, cancerous parts in the testicle, skin, and other places.

Such

Such is the nature of this work, of which we have given a very ample detail. The subject is highly important, and the advantages Mr. Home has enjoyed in so long a communication with Mr. Hunter, induced us to expect much information from him. If we have been disappointed, we impute it rather to the intricacy of the subject than any deficiency of ability or industry in the author.

---

*A concise and systematic Account of a painful Affection of the Nerves of the Face; commonly called Tic Douloureux. By* S. Fothergill, M. D. *Physician to the Western Dispensary.* 12mo. pp. 105.

The *Tic Douloureux, Trismus Dolorificus* of Sauvage, or, as the author of the Treatise before us terms it with more accuracy, *Faciei Morbus Nervorum Crucians,* is perhaps the most painful chronic complaint with which we are acquainted; and though comparatively rare, practitioners have every now and then the misfortune to meet with it, and to deplore the intense sufferings of the patient, and the lamentable inefficacy of the common resources of art.

The written descriptions of this disease are few and of modern date. Dr. Fothergill, uncle to the author of the present treatise, was the first who distinctly brought it into notice in England; since which time a more detailed and laboured history of the disease has been published in France, by M. Pujol, entitled, *Essai sur la Maladie de la Face*; a learned paper with comments on Pujol, by Thouret, inserted in the Mém. de la Societé Royale de Medicine; a few detached remarks on the subject, with cases, in some foreign Journals; and lastly, but certainly one of the highest in value, Dr. Haighton's succinct comprehensive paper, inserted in the Medical Records and Researches, containing also the proposed plan of cure by division of the nerve.

From these authorities, and, as appears, from some personal experience, Dr. F. has drawn up, with judgment and fidelity, the present monographic description.

The several ramifications of the second branch of the fifth pair of nerves are the chief parts affected by this terrible disease. The author's more detailed description is the following.

" The most frequent seat of the affection, is the nerves over the os malæ, just below the orbit; the ala nasi, upper lip, teeth, and gums. When this is the case, it will be found to proceed from the second branch of the fifth pair of nerves, the *superior maxillary nerve,* which passes through the foramen rotundum, and whose branches are chiefly distributed to those parts. Sometimes the forehead and temple, and inner canthus of the eye, and even the globe of the eye itself, are first affected, from the first branch of the fifth pair, the ophthalmic branch being the subject of the disease; and as there are some cases recorded in which the patient suffered much from an effusion of scalding tears, it might probably arise from that branch of the ophthalmic, which goes to the lachrymal gland, being affected. The two other chief branches of the ophthalmic thalmic, which are, however, very rarely the seat of this affection, are the frontal and the nasal; the first of which is distributed to the muscles surrounding the eye, and the muscles and integuments of the forehead; whilst the nasal branch passes obliquely through the orbit, giving off one or two twigs to the fasciculi of the ciliary nerves; and then is continued betwixt the superior oblique and adductor muscles, passes through the internal orbital foramen; and, after again entering the skull, passes once more out of the cranium through the cribriform plate of the ethmoid bone, to be finally distributed to the superior spongy bones, and frontal sinuses: when, in addition to the parts already named, the lower jaw and tongue are affected, the third branch of the fifth pair, or lower maxillary nerve, is diseased. Perhaps, as frequently as any of these nerves, is the portio dura of the seventh pair diseased; it gives off branches to most parts of the face, and, from its spreading, is named pes anserinus. Its branches communicate with several of those of the fifth pair. The distinguishing mark of its being affected is, that besides the parts already enumerated, we find pain in the ear, the mastoid psocess, and the angle of the lower jaw. The disease then is chiefly confined to the fifth pair of nerves, of which most frequently the second branch only is affected, and the branches of the portio dura of the seventh pair. But, from the intimate connexion of most of the branches of these nerves with each other, the disease seldom continues long without extending its ravages; and, as there are communications more or less intimate between, I believe all the first seven pair of nerves, which are distributed to every part of the head and face; it is just to suppose that, in very inveterate cases, all the nerves may be affected."

The description of this disease is necessarily defective in two great points, the predisposing and the proximate causes, for since the indefensible hypothesis (or rather only conjecture) of the celebrated Dr. Fothergill of a cancerous acrimony, not a plausible opinion of the cause has been advanced. The author does not add to the number of conjectures, but indulges himself in some very trite invectives against speculative reasoning and futile hypothesis on medical subjects, which, with singular infelicity of observation, he asserts to be the peculiar folly of the present day; an assertion which the least insight into the history of medical opinions would shew to be utterly void of foundation.

The diagnosis is important, not so much to insure the right treatment of this disease (as unfortunately it baffles every resource of medicine) as to prevent the patient from undergoing the additional pain and trouble of the remedies used in the disorders with which it may be confounded.

The most vexatious of these mistakes is for the tooth-ach, as it leads the practitioner to propose, and the patient to suffer (for he will generally submit to any thing in this excruciating disorder) the loss of many sound teeth with no benefit whatever. We shall transcribe part of the author's diagnosis.

"From

"From odontalgia, then, it is known by the age of the patient; the morbus crucians seldom appearing before that period of life when the tooth-ach is least prevalent, and when there are often very few teeth remaining. But what is chiefly to be relied upon, and which peculiarly distinguishes it from all other affections, is the shortness of the paroxysm, and the rapidity of its succession; and during the interval, an entire freedom from all pain; the seat of the pain, and its darting in several directions, according to the particular nerve affected, with an acuteness and poignancy differing from that of the tooth-ach, which seem to strike deep, whilst the pain of the morbus crucians is always more superficial, and infinitely more lancinating; and lastly, the convulsive twitchings, which, though not always present, are very frequent, and are never experienced in odontalgia.

"From rheumatism it is distinguished by a paroxysm being excited by the slightest touch; by the shortness of its duration, and the extreme violence of the pain: neither are the symptoms similar; for in rheumatism, if acute, there is general fever, redness and increased heat in the affected part, and generally swelling; if chronic, the pain is dull, obtuse, long continued, and often increased at night; but none of these symptoms occur in the morbus crucians. When rheumatism and tooth-ach are combined, the symptoms are equally distinct. In such cases there is rarely an interval of ease, and the night is generally the period when the pain is most troublesome.

"From syphilitic pains, the disease is readily known by their history; their great alleviation during the day; their increased violence when warm in bed; and by their uniformly yielding to the use of mercury.

"From gout, the morbus crucians is easily distinguished, by the patient passing in a moment from perfect ease to the utmost torture, which again as suddenly ceases; and there are many symptoms in gout, that are not present in the disease we are treating of: indeed, I only know of one in common with both, and that is pain; but even this differs sufficiently, to prevent the least difficulty in discriminating between the two diseases."

The painful disease termed *hemicrania* appears to be more of a rheumatic nature, though nosological writers are not quite agreed as to its symptoms. If hemicrania be considered simply as an intermitting rheumatic pain of the face, the tic douloureux, which resembles it in many particulars, may be clearly distinguished by the circumstance of the pain, in the latter disease, accurately following the ramifications of the affected nerve; so that, as Dr. Haighton has well observed

"An unintelligent patient will frequently describe the dartings of pain so minutely, as to teach the practitioner the distribution of the several branches of the fifth pair, if he had not known it before."

Under the prognosis, Dr. F. introduces some good practical observations by Dr. Curry, Physician to Guy's Hospital,

In a well-marked case, here given on the authority of the late Dr. Cappe, of York, the following curious fact is added, shewing the power of fixed attention of the mind on a favourite object (independent of any sudden or energetic impression) to overcome a most acute bodily pain.

"A singular circumstance attending this patient, was, that when the pain was very intense, if he resolutely sat down to play at whist, a game of which he was particularly fond, the pain would generally cease, before he had played a single rubber; and whilst his attention was completely absorbed in the game, not another paroxysm occurred.

"This happened too often to be considered as accidental."

Little is to be added, on the subject of the treatment of this disease, to the observations contained in Dr. Haighton's paper. The operation of dividing the nerve is fully justified by the extreme acuteness of the disorder, and by the considerable degree of success that has attended this mode of cure. It is well known that this operation, though a radical cure in the part immediately affected, does not always prevent a recurrence of the pain in collateral branches of the nerves.

How far the pain may be safely followed up by the knife, and when it would be prudent to change the exterminating for the palliative plan of treatment, is yet doubtful, and must besides be determined by the circumstances of the individual case.

---

*The Popular Compendium of Anatomy, or, a concise and clear Description of the Human Body, with the Physiology, or Natural History of the various Actions and Functions of its different Organs and Parts; containing also an Article on suspended Animation, with the proper Means to be used for the Recovery of drowned Persons.* By WILLIAM BURKE, Surgeon. 12mo. pp. 261. London, 1804.

As much real knowledge in Anatomy and Physiology as the student will acquire, and as much curiosity as will be excited in the general reader, by the perusal of any brief compendium assisted by two or three minute engravings, may be fairly expected from a selection like the one before us, correct, sufficiently well distributed, intelligible, and in all respects very much to the purpose. An analysis of its contents would be idle, the following will serve as a specimen.

"Amphibious animals can live long without respiring; they exhibit a less complicated system for the function, they have no ribs, no diaphragm, no chest in fact. The respiration of frogs will illustrate this; they breathe through the nostrils, and distend their throat with air; the muscles of the throat force it into the lungs, which are emptied by a slight motion of the sides of the creature. So that they swallow the air by their broad extended jaws, and come to the surface of the water to procure it only occasionally.

"The

"The lungs in these animals, and in lizards, the camelion, and even the crocodile, who all breathe in the same manner with the frog, are thin, delicate, transparent bags, with but few arteries and veins distributed upon them; which is the reason that they require the influence of the air in a slender degree only.

"Fishes again have a mode of respiration peculiar to themselves. They breathe air when mixed with water, and this office is performed by their gills. These are formed by tender membranous fringes, through every part of which the blood circulates. They are protected by broad flaps, which move, as it were, upon hinges. These fringes are separated when the fish opens its mouth and drives the water backwards by a motion of its jaws; so that it is forced between each feathery extremity of the red gills. Here the blood is exposed to the water and the air which is dissolved in it; for fishes can no more live in water deprived of its air by boiling, or by an air-pump, than man or quadrupeds can live in a vacuum. If put into such water, they rise to the surface and gasp for air. Fishes cannot breathe in air alone, for that element is not accommodated to the mechanism of their lungs.

"The breathing of fishes unfits water for that function, as air is corrupted by the respiration of man; if we separate the air from the water in which fishes have lived, it is no longer the same air it previously was, and it is contaminated precisely in the same way as air is which has been respired by man. Hence it is that fishes when confined in a small vessel rise to the surface, and that in frozen ponds they crowd around the holes which are made in the ice. Even shell-fish have a sort of gills for the performance of this function; and the beard of the oyster is a curious and admirable example of their structure.

"The last species of animal respiration we shall notice is that of insects. They have no breathing organ like the lungs, but their bodies are pierced with air tubes in every direction. They divide very minutely like the delicate vessels of leaves and flowers, and penetrate every part of the animal; but the objects are so small, and the changes of form which insects assume are so rapid, that we cannot point out all the uses of this peculiar structure."

---

### *Mr. Wilkinson's Elements of Galvanism.*

( Concluded from pp. 82—87. )

A detailed Journal of Galvanism, collected with much more industry than arrangement, is not a subject for analysis. The discovery of the voltaic pile opens the second volume; and the vast mass of experiments to which this beautiful fact in natural philosophy has given rise, are detailed with sufficient minuteness. The author is known to possess a very extensive apparatus of Galvanic troughs; and the extreme brilliancy of many of the experiments which it enables him to perform, has been witnessed by numerous spectators. The deflagration of metals is attended with peculiarly beautiful appearances, and on this subject the author gives the following remarks:

"I have been given to understand, that it was with the celebrated electrical apparatus at Haerlem, made by Mr. Cuthbertson, that the beautiful metallic oxyds were first struck upon paper, each of the metals having produced its peculiar characteristic tint.

"A powerful charge of electricity oxydates a considerable length of metallic wire; while galvanism acts on successive portions of the metal, whenever it is excited by a conducting medium. Electricity produces all its effects by one sudden and violent discharge; while galvanism operates by its continual current. It is on this account, that the sensations produced by their diverse actions, are materially different. The electric shock, from a very small jar, operates on the body by a sudden and percussive effect; while the one which follows the galvanic process, seems to arise from a constant current, attended by a jarring and tremulous sensation.

"All finely drawn metallic wires are rapidly burned by means of a large galvanic battery, the powers of which are in general ascertainable by the extent of fine steel wire, such as the pendulum springs of watches, which the battery will instantly render red-hot. This constitutes the most correct *galvanometer;* while the other, on which I have already touched, ought to be termed a *galvanascope*. A powerful battery, such as the one I generally employ, will induce a red heat on several inches of the above steel wire; and in this way I am enabled to ascertain whether my battery is in proper order.

"To deflagrate finely laminated metals, good charcoal is the best medium which can be employed. As the substance of the metallic leaves, when laminated, is much thinner than that of any wires which can be drawn, the deflagrations are more brilliant, and the light extremely vivid. Some metals exhibit a different appearance from others.

"Gold leaf, having the thickness of the $\frac{1}{282000}$ part of an inch only, is not merely deflagrated, but is likewise completely oxydated, and reduced to a purple powder.

"Silver leaf, which is thicker than gold leaf in the proportion of 7 to 4, is likewise converted into an oxyd, and exhibits a beautiful greenish light. We thus see, that by the powers of galvanic electricity two metals, which resist the utmost force of our most powerful furnaces, are rapidly oxydated.

Copper leaf, or, to speak more correctly, brass leaf, being the yellow Dutch metal made from copper plates, by cementation with calamine without a subsequent fusion, has a thickness nearly five times greater than that of gold leaf. In its deflagration, red ignited particles of copper are detached, on this account, that copper requiring a very high temperature before it fuses, the ignition takes place first. The copper, before it melts, is constantly red-hot, red-hot, which is not the case with tin, lead, &c. The Dutch silver leaf, a composition of tin and zinc, about ten times as thick as gold leaf, is deflagrated with rapidity.

"Tin-foil, which is also a composition of tin and zinc, is about 300 times thicker than gold leaf. Narrow slips of this substance are also easily deflagrated. In short, all metals, if in a due state of tenuity, are capable of being deflagrated.

"When metals are placed under an exhausted receiver, they give out light, but are not oxydated.

"When metallic leaves are deflagrated in carbonic acid gas, the light is not vivid; but in oxygen gas, the contact is no sooner established, than the metallic leaves are destroyed with one sudden flash.

"Brugnatelli has conjectured, that the action of galvanism, in the decomposition of water, produces a compound which he apprehends to result from the union of the disengaged electricity with the oxygen, and which he terms an electrat. This electrat is capable of dissolving metallic substances. Thus, with copper, it forms a beautiful green; with zinc, a dark grey; and with iron, a reddish yellow oxyd. These effects, as I have already observed, appear to arise from the combination of nitrous acid and ammonia. They are probably produced in every galvanic decomposition of water.

"Van Marum, in making the circuit through quicksilver, noticed a beautiful effect. The end of a fine wire having been made to form the communication, a powerful combustion ensued, which dispersed the mercury on all sides, and sparkles, having the appearance of thousands of rays, constituting fine suns of several inches in diameter, were produced."

The author then attempts, by reasoning and by some experiments, to invalidate Dr. Crawford's theory of respiration and animal heat, and even to shew that respiration is not the cause of animal heat. The imperfections of Dr. Crawford's hypothesis, and the valid objections to which some of his experiments are liable, are well known to those who have attended to this branch of chemical physiology; but it is not by such inconclusive arguments and unsatisfactory experiments as the author brings forward, that the system of Crawford and Jurin will be overturned.

A few remarks are added on the medical powers of galvanism. As these are given without parade and exaggeration, and with all the appearance of candour and fidelity, we shall extract a few of them. The following relate generally to the application of galvanism.

"In a case of hemiplegia, as before mentioned, a piece of gold leaf having been applied on the right side of the forehead, and another on the arm of the left side, as often as the circuit is completed, the arm becomes convulsively agitated. During the operation, one of the conductors should be left in contact with one of the pieces of metallic leaf, while the other conductor, which is employed to complete the circuit, should be removed immediately after the contact is made. The operator should proceed thus for about ten minutes or a quarter of an hour, according to the nature of the case, and the degree of inflammation induced on the parts.

"Very soon after the application of galvanism, an areolous redness is perceived; and if it be persevered in too long, vesications and subsequent ulcerations are produced. These symptoms, which are a little troublesome for the moment, do not require any particular treatment in their cure. The part of the body to which the conducting wire from the copper side of the battery is applied, is always the most powerfully acted on; and if the conducting wires be kept in contact with the metallic leaves, for the space even of half a minute, without being removed, the one from the copper side will produce an acute pricking sensation, very much resembling the bite of a leech.

"In some cases, attention ought to be paid to this difference between the ends of the battery. When it is wished to act on one part of the body more powerfully than on the other, the conducting wire from the copper end should be placed on that particular part. This difference in power corresponds with the effects experienced from the charged Leyden jar, in the case of which, the part of the body connected with the negative side of the jar is more powerfully acted on than the part connected with the positive side. In recent cases of hemiplegia, very good effects are soon perceived. After a few applications, a sensation of returning warmth is felt; and the action of the muscles of the arm restored, as often as the circuit is completed. After the operation, the use of the flesh-brush, persevered in for about a quarter of an hour, contributes to re-establish the circulation of the diseased parts."

Paralytic affections, either general or local, have always been thought peculiarly to demand the trial of electricity, as a powerful stimulus, whose most active operation appears to be upon the nervous system. Galvanism probably stands on the same ground with electricity in this respect, and certainly deserves a trial. The author gives his testimony to its partial efficacy, though in no very high terms. In that species of deafness, which arises from apparent defective energy in the auditory nerves, galvanism seems to have produced very good effects. The author's mode of application is very effectual. It can be properly understood only by consulting the plates. In diseases of the eyes, and incipient amaurosis, which would appear peculiarly fitted for the application of this remedy, no success has been obtained. The following testimony is more favourable:

"In involuntary actions of the muscles, I know of no remedy so efficacious as galvanism. In a contracted state of the fingers, or hands, however violently the latter may be clenched, on the application of this principle for the space of a few minutes, it rarely rarely fails to induce a relaxation. In cases of cramp, if of long continuance, and even of tetanus, or locked jaw, it has afforded relief in a short space of time. In contractions of the joints, and in all cases of rigidity, it will be found a very advantageous stimulus, which will greatly contribute to the restoration of motion.

"In the stiffness of the joints, occasioned by the gout, it has come under my observation that the stimulus of galvanism, conjointly with the flesh-brush, has been attended by the happiest effects. It seems to give such a tone to the blood-vessels as to render the circulation more vigorous. In this state of the disease such an effect is very desirable.

"Perhaps, in no case are the advantages of galvanism more sensibly experienced, than in indolent tumours, or scrophulous swellings, which have long remained stationary. By the influence of this principle, tumours of this kind have in a few days been brought either into a state of suppuration or resolution. Many swellings are of such a nature, that their removal by either of these means are desirable. I have frequently applied the galvanic principle with the utmost success in inguinal tumors, which had resisted every other curative intention. The obtuse aching sensation, generally attendant on these indolent tumors, is very speedily removed. In scrophulous affections of the neck, it has been found very beneficial."

In cases of asphyxia, from any cause, galvanism promises to become an agent of infinitely more power in restoring life than any hitherto known. The astonishing experiments of Aldini on mutilated and decapitated animals, exhibit the extreme energy of this principle in restoring all the vital functions; and this, assisted by the judicious expansion of the lungs by inflation, may perhaps, give us a latitude in restoring suspended animation, beyond the most sanguine expectations of the most zealous philanthropist. In this point of view, galvanism certainly merits the most persevering research. The author of the volumes before us has shewn himself equally as well qualified to pursue the science as to impart his knowledge to others. We heartily wish him success in this new and interesting branch of natural philosophy.

